# Development of a questionnaire to assess the collaboration between clinicians from different levels of care

**Published:** 2012-09-04

**Authors:** Nuria Toro Polanco, Roberto Nuño Solinis, Regina Sauto Arce, Iñaki Berraondo Zabalegui, Leticia San Martín Rodríguez

**Affiliations:** O+Berri, Basque Institute for Healthcare Innovation, Spain; O+Berri, Basque Institute for Healthcare Innovation, Spain; O+Berri, Basque Institute for Healthcare Innovation, Spain; Hospital Bidasoa, Osakidetza (Basque Health Service), Spain; Department of Professional Development and Training in Nursing, University of Navarra Clinic, Spain

**Keywords:** interprofessional collaboration, questionnaire, integrated care

## Abstract

**Purpose:**

The purpose was to develop a questionnaire, gathering the professionals’ opinion, to measure the degree of collaboration between clinicians from different levels of care (primary care and specialised care) in a given healthcare organisation. This questionnaire was originally developed to assess processes of care integration in the Basque Health System (Spain), but can also be used in other contexts.

**Methodology:**

The questionnaire was based on the four-dimension model and 10 indicators of interprofessional collaboration in healthcare organisations proposed by D’Amour et al. [1].

The resulting questionnaire was pre-tested and its construct validity and homogeneity evaluated in three healthcare organisations of the Basque Health Service.

**Results and conclusions:**

A 10 items questionnaire has been developed. Each of its items corresponds to one of the indicators proposed by D’Amour et al. [1]. The title of each of these items is: 1) Shared goals, 2) Patient-centred approach, 3) Mutual knowledge, 4) Trust, 5) Strategic guidelines, 6) Shared leadership, 7) Support for Innovation, 8) Forums for meeting, 9) Protocolisation and 10) Information Systems. Clinicians are asked to value each of these aspects in their healthcare organisation on a Likert scale of 5 degrees.
